# A Pilot Proteogenomic Study with Data Integration Identifies MCT1 and GLUT1 as Prognostic Markers in Lung Adenocarcinoma

**DOI:** 10.1371/journal.pone.0142162

**Published:** 2015-11-05

**Authors:** Paul A. Stewart, Katja Parapatics, Eric A. Welsh, André C. Müller, Haoyun Cao, Bin Fang, John M. Koomen, Steven A. Eschrich, Keiryn L. Bennett, Eric B. Haura

**Affiliations:** 1 Department of Thoracic Oncology, H. Lee Moffitt Cancer Center and Research Institute, Tampa, Florida, United States of America 33612; 2 CeMM Research Center for Molecular Medicine of the Austrian Academy of Sciences, Lazarettgasse 14, 1090 Vienna, Austria; 3 Cancer Informatics Core Facility, H. Lee Moffitt Cancer Center and Research Institute, Tampa, Florida, United States of America 33612; 4 Department of Biostatistics and Bioinformatics, H. Lee Moffitt Cancer Center and Research Institute, Tampa, Florida, United States of America 33612; 5 Proteomics Core Facility, H. Lee Moffitt Cancer Center and Research Institute, Tampa, Florida, United States of America 33612; 6 Department of Molecular Oncology, H. Lee Moffitt Cancer Center and Research Institute, Tampa, Florida, United States of America 33612; Institute of Biomedical Sciences, TAIWAN

## Abstract

We performed a pilot proteogenomic study to compare lung adenocarcinoma to lung squamous cell carcinoma using quantitative proteomics (6-plex TMT) combined with a customized Affymetrix GeneChip. Using MaxQuant software, we identified 51,001 unique peptides that mapped to 7,241 unique proteins and from these identified 6,373 genes with matching protein expression for further analysis. We found a minor correlation between gene expression and protein expression; both datasets were able to independently recapitulate known differences between the adenocarcinoma and squamous cell carcinoma subtypes. We found 565 proteins and 629 genes to be differentially expressed between adenocarcinoma and squamous cell carcinoma, with 113 of these consistently differentially expressed at both the gene and protein levels. We then compared our results to published adenocarcinoma versus squamous cell carcinoma proteomic data that we also processed with MaxQuant. We selected two proteins consistently overexpressed in squamous cell carcinoma in all studies, MCT1 (SLC16A1) and GLUT1 (SLC2A1), for further investigation. We found differential expression of these same proteins at the gene level in our study as well as in other public gene expression datasets. These findings combined with survival analysis of public datasets suggest that MCT1 and GLUT1 may be potential prognostic markers in adenocarcinoma and druggable targets in squamous cell carcinoma. Data are available via ProteomeXchange with identifier PXD002622.

## Introduction

Recent developments in liquid chromatography and high-resolution mass spectrometry (MS) have allowed for highly precise, comprehensive characterization of proteomes [1]. Proteogenomics, which combines discovery proteomics with genomic technologies, has emerged as an important tool in the understanding of the complexities of the tumor genome and how they are integrated into a functional proteome [2]. Groups such as the Clinical Proteomic Tumor Analysis Consortium (CPTAC) are taking advantage of these advances and setting the bar high with thorough proteogenomic characterizations of a large numbers of tumor samples (i.e., cohorts of ~100 patients) [3]. There are several computational challenges to performing proteogenomic studies [4]. New tools and pipelines have been developed to tackle these problems, but a one-size-fits-all solution remains elusive [5–9].

Sample throughput is an issue in MS-based discovery proteomics because the necessary biological and technical replicates easily generate dozens of samples. These numbers can expand significantly when analyzing large groups of patient samples in order to develop specific biomarkers or molecular classification schemes, and these numbers can expand even further if samples are fractionated using chromatographic methods in order to increase the depth of proteome coverage. One solution to this increasing sample number problem is to utilize multiplexing methods with isobaric chemical labeling reagents [e.g., tandem mass tag (TMT) or isobaric tags for relative and absolute quantitation (iTRAQ)]. These methods provide a means for parallelization by running many samples simultaneously in a single mass spectrometer [10].

Proteomic methods are often being used to comprehend the underlying biology of lung cancer and to identify potential biomarkers [11], and proteomic profiling is showing promise for clinical use [12,13]. Proteomic comparisons of two prominent non-small cell lung cancer (NSCLC) subtypes, adenocarcinoma (ADC) and squamous cell carcinoma (SCC), have been previously reported using label-free quantification and SILAC [14–17], and some proteogenomic studies of ADC and SCC have also been undertaken [18,19]. Our first goal was to understand how a TMT-based proteogenomics study would perform in our hands experimentally and to determine whether special considerations would arise while analyzing TMT-derived data. If our results were promising, then our next goal was to use a TMT-based proteogenomic approach, moving forward with a larger study with more patient samples. Here, we undertook a pilot proteogenomic study of three ADC and three SCC tissue samples using 6-plex TMT and a custom Affymetrix microarray [Gene Expression Omnibus (GEO) no. GPL15048]. MaxQuant was used to identify peptides and to extract and quantify reporter ion intensities for comparison of ADC and SCC tissues [20]. We then compared our results to previously published studies that used different discovery proteomic strategies in order to identify prevalent candidate biomarkers (see Table 1).

**Table 1:** NSCLC Proteomics Summary.

	Stewart et al.	Kikuchi et al. [15]	Li et al. [19]
**Replicates**			
ADC samples	3	4 (20)*	7
SCC samples	3	4 (20)*	4
Quantification	6-plex TMT	label-free	label-free
Fractions	50	20	1
Total MS run time	98 min	95 min	300 min
**Intensities (log**_**2**_ **)**			
Type	Reporter Ion	Peptide	Peptide
Mean	11.4	21.55	17.72
Median	11.5	21.84	18.94
Standard deviation	1.43	3.11	4.42
Minimum (non-zero)	4.42	15.94	10.02
Maximum	17.57	34.15	31.27
**Peptides**			
Unique peptides	51,001	18,198	39,048
Missing peptide intensities	395 (0.1%)	23,287 (16%)	92,255 (21%)
Mean peptide length	12.26	13.58	15.99
Mean posterior error probability score	0.0057	0.0046	0.0070
**Proteins**			
Unique proteins	7,241	4,850	4,562
Single peptide protein identifications	1527 (21%)	1492 (31%)	752 (16%)
Peptides per protein	7.04	3.75	8.56
**Differential Proteins (ADC vs SCC)**			
with *P* < 0.05	565	1,528	244
with *P* < 0.05 and fold-change > 1.5 or < 0.6667	279	1,465	231
with *P* < 0.01	113	869	66
with *P* < 0.001	11	431	11

*Kikuchi et al. used 4 pools of the same 20 samples in their analyses.

## Materials and Methods

### Sample Preparation for Liquid Chromatography-Mass Spectrometry

This study was approved by Liberty IRB Inc., an independent review board company, under Institutional Review Board approval number 12.11.0023. Tissue samples for this analysis were from patients who had provided prospective written informed consent to be included in Moffitt’s Total Cancer Care^®^ (TCC) institutional protocol. Quantitative proteomic analysis was performed on tumor tissues isolated from six individual patients: three diagnosed with lung ADC and three with SCC (S1 Table). To aid tissue lysis, frozen samples were macerated in a tissue pulverizer. The pulverizer was then rinsed with 2 mL water, and each sample transferred to a 2-mL Eppendorf tube to lyse erythrocytes. Samples were centrifuged at 5,000*g* for 2 min, and the supernatant was removed. Lysis buffer (1.0 mL of 50 mM HEPES, pH 8.5, supplemented with 2% SDS) was added, and the samples were incubated for 20 min at room temperature. The samples were then heated for 5 min at 99°C and sonicated (Covaris) to reduce viscosity. After centrifugation at 16,000*g* for 15 min, the supernatant was collected. The protein amount in each sample was determined by using the bicinchoninic protein assay (Pierce Biotechnology/ThermoFisher Scientific, Waltham, MA) followed by an adaptation of the filter-aided sample preparation method [21,22]. Three samples each of ADC and SCC (100 μg of lung tissue lysate) were reduced with 100 mM dithiothreitol at 99°C for 5 min and transferred into VIVACON 500 filter units (Vivaproducts Inc., Littleton, MA). SDS-containing buffer was removed from the sample by centrifugation and exchanged with 8 M urea in 100 mM Tris-HCl buffer. Proteins were alkylated with 50 mM iodoacetamide and washed with 50 mM triethyl ammonium bicarbonate. Porcine trypsin (Promega Corp., Madison, WI) was used to digest the proteins in an enzyme-to-protein ratio of 1:100 (wt/wt).

For relative protein quantitation, the six samples were separately derivatized with 6-plex TMT reagents (ThermoFisher Scientific), according to the instructions provided by the manufacturer. The ADC and SCC samples were labeled with TMT 126, 127, 128 and TMT 129, 130, 131, respectively. The labeled tryptic digests were pooled and concentrated by solid-phase extraction (MacroSpin columns 30–300 μg capacity; The Nest Group Inc., Southborough, MA). Samples were basified with 20 mM ammonium formate prior to injection onto a reversed-phase column (150×2.0 mm Gemini^®^NX-C18 3 μm 110Å; Phenomenex, Torrance, CA) using an Agilent 1200 series high-performance liquid chromatography (HPLC; Agilent Biotechnologies, Palo Alto, CA) with ultraviolet detection at 214 nm with solvent A (20 mM ammonium formate, pH 10, in 5% acetonitrile) and solvent B (20 mM ammonium formate, pH 10, in 90% acetonitrile). Peptides were separated at a flow rate of 100 μL/min and eluted from the column with a linear gradient from 0% to 70% solvent B. Seventy-two time-based fractions were collected into a 96-well plate, with the first 18 and last 7 fractions pooled into 3 and together with all other fractions transferred to a total of 50 HPLC vials. Samples were acidified with 5% formic acid, organic solvent was removed in a vacuum concentrator at 45°C, and peptides were resolubilized and diluted in 100 to 400 μL of 5% formic acid, depending on the intensities of the individual ultraviolet traces [23]. Individual fractions were analyzed at pH 2.4 on an Agilent 1200 nano-HPLC system (Agilent Biotechnologies, Palo Alto, CA) coupled to a hybrid linear trap quadrupole Orbitrap Velos mass spectrometer (ThermoFisher Scientific) utilizing the Xcalibur software version 2.1 for data acquisition. Single fractions were loaded onto a trap column (Zorbax 300SB-C18 5 μm, 5 × 0.3 mm; Agilent Biotechnologies, Palo Alto, CA) with a binary pump at a flow rate of 45 μL/min. Loading and washing solvents were composed of 0.1% trifluoroacetic acid in water (solvent A) and 0.1% trifluoroacetic acid in 70% methanol and 20% isopropanol (solvent B). The peptides were eluted by back-flushing from the trap column onto a 16-cm fused silica analytical column with an inner diameter of 50 μm packed with C18 reversed-phase material (ReproSil-Pur 120 C18-AQ, 3 μm, Dr. Maisch GmbH, Ammerbuch-Entringen, Germany). LC-MS solvents were composed of 0.4% formic acid in water (solvent A) and 0.4% formic acid in 70% methanol and 20% isopropanol (solvent B). Elution was achieved with a 27-min gradient ranging from 3% to 30% solvent B, followed by a 25-min gradient from 30% to 70% solvent B and, finally, a 7-min gradient from 70% to 100% solvent B at a constant flow rate of 100 nL/min.

The analysis was performed in data-dependent acquisition mode. The 10 most intense ions were isolated and fragmented by higher-energy collision-induced dissociation (HCD) for peptide identification and relative quantitation of TMT reporter ions. Dynamic exclusion for selected ions was 60 sec and a single lock mass at *m/z* 445.120024 (Si(CH_3_)_2_O)_6_) was used for internal mass calibration.[24] The maximally allowed ion accumulation time was set to 500 and 200 ms for MS^1^ and MS^2^ scans, respectively. Overfilling of ion traps was prevented by automatic gain control set to 10^6^ ions for a full Fourier transform MS scan and 5×10^5^ ions for MS^2^ HCD scans. Intact peptides were detected in the Orbitrap mass analyzer at a resolution of 30,000 with a signal threshold of 2,000 counts for triggering an MS/MS event. HCD-MS^2^ spectra were acquired with 1 microscan at a resolution of 7,500. The mass spectrometry proteomics data have been deposited to the ProteomeXchange Consortium via the PRIDE partner repository with the dataset identifier PXD002622 [25].

### MaxQuant

Raw files from the Kikuchi et al. study were obtained directly from the authors, and raw files from the Li et al. study were obtained from ProteomeXchange (http://www.proteomexchange.org/) [25]. Raw files were grouped and analyzed by study using MaxQuant version 1.5.1.2. Peaks were searched against the UniProt human database (20,193 sequences; released August 2014; http://www.uniprot.org) with the Andromeda search engine [26]. When possible, all raw files were processed using similar parameters; however, workflow-specific changes were needed: the TMT data were processed by setting the “type” parameter to “reporter ion” and selecting the relevant TMT 6-plex settings. At least seven amino acids per peptide were required, and as many as two missed cleavages were allowed. A false discovery rate of 0.01 was used for both peptides and proteins. The “match between runs” option was selected using a time window of 4 min. N-terminal acetylations or methionine oxidations were both modifications allowed in protein quantification. MaxQuant results were manually spot-checked using the MS browser, OpenChrom [27].

### Affymetrix Gene Microarray

Tissues were processed and assessed for RNA quality according to the TCC protocol prior to expression analysis. The six lung tumor samples were profiled using a custom Affymetrix GeneChip that measures the expression of 60,607 distinct transcripts (GEO GPL15048). All probes with standard deviation > 1 (11,008 or 18% of total probes) were used for the gene expression heat map.

### Peptide and Protein Data Processing

Peptide intensities were extracted from MaxQuant output and input into Libaffy [28]. Libaffy consists of a set of routines for accessing the various file types and post-processing them using a variety of algorithms, including iterative rank-order normalization (IRON) [28–30]. Peptide intensities were normalized using IRON with proteomic parameters and then input into R/RStudio with corresponding metadata [31,32]. Peptides were filtered for posterior error probability > 0.1, reverse sequences, non-human contaminant peptides, and missingness. Peptides were excluded from further analysis if there were more missing values than the larger of the two NSCLC sample sizes. Remaining peptide intensities were log_2_-transformed and mapped to possible protein matches.

In the event that peptides mapped to multiple proteins, called the protein inference problem, MaxQuant will choose the protein from a set of peptides based on which protein has the most matching peptides and would thus be perceived to be the most abundant [33]. Constituent peptide intensities are then summed in MaxQuant to give the resulting protein intensity. The major drawback to this approach is that the next most abundant protein or proteins, even if they are very close to the abundance of the first protein, will not be included in the results. Additionally, the authors of MaxQuant point out that selecting the protein with the most peptides might not always be the best decision [34]. Furthermore, the process of summing the peptide intensities to obtain the protein intensity can artificially lower the final protein intensity in the case of missing data, and this can be a pervasive issue since proteomic data are rife with missingness [35]. We implemented a more liberal peptide-to-protein mapping methodology to address these issues. We began by mapping peptides to all possible proteins, and we then took the weighted mean (also known as Tukey’s biweight; implemented in the R Bioconductor package “affy”) to obtain a protein intensity that is more robust than the summation of intensities [36–38]. The weighted mean penalizes outlier values in its calculation so it will more closely reflect the true intensity values for a particular protein. A Welch two-sample T-test was used on the resulting protein intensities to ascertain differentially expressed proteins between ADC and SCC.

We generated mapping tables using data from International Protein Index, UniProt, and Genbank to match the microarray probes to proteins. If a protein had multiple matching probes, then we chose the probe with the highest average intensity to use as the gene expression. Because our goal was to compare the gene expression to the protein expression, we filtered to only include results that were in both the protein expression dataset and the gene expression dataset.

### Statistical Analyses

Concordance of gene and protein expression was measured using both Pearson correlation (herein referred to as *R*) and Spearman correlation (herein referred to as *ρ*). Clustering was performed using Pearson correlation as a similarity measure and complete linkage to create the dendrogram for the heat maps. Welch two-sample, two-sided T-tests were used to compare expression of genes and proteins. In general, a significance level of 0.05 was used for statistical testing, and we reported the P value or significance level any time a statistical test was performed. Unless otherwise noted, *P* values were not adjusted for multiple hypothesis testing.

We used KM Plotter (http://www.kmplot.com) to perform survival analysis of MCT1 and GLUT1 in ADC and SCC [39]. This tool facilitates combined survival analysis across multiple microarray datasets including The Cancer Genome Atlas (TCGA), GEO, and caArray. Data was normalized using the MAS5 algorithm and a second scaling normalization was used to reduce batch effects. Only probes that are in common across all datasets were used. KM Plotter provides a user-friendly interface to the R programming language (“survival” Bioconductor package) to perform univariate and multivariate Cox regression (including estimating hazard ratios), generate Kaplan-Meier curves, and calculate logrank *P* values. KM Plotter was accessed March 2015 for the univariate analyses and October 2015 for the multivariate analyses.

## Results

### Quantitative Proteomics Identifies Differences Between NSCLC Subtypes

**Fig 1:**
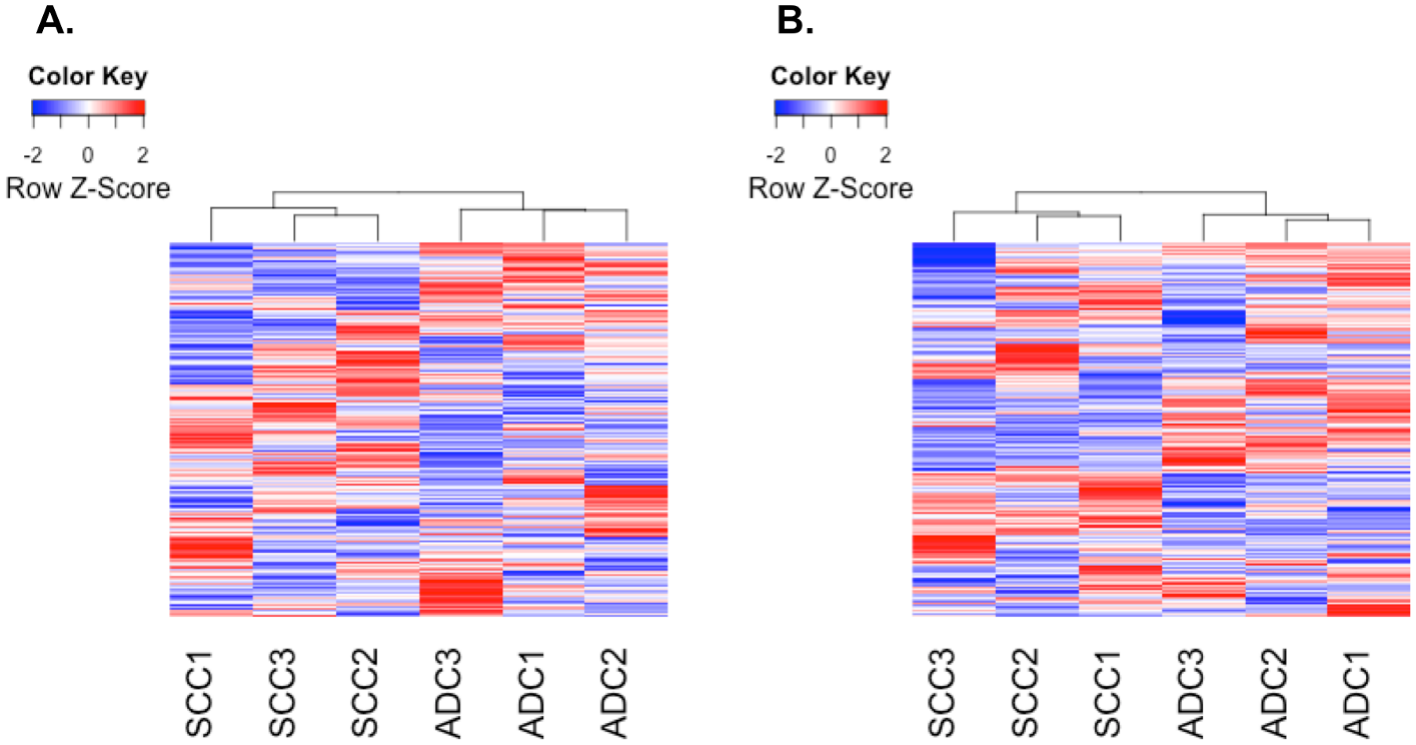
Proteogenomic Data Recapitulates NSCLC Histology. (A) Clustering of all identified proteins (7,241) from quantitative TMT analysis group tissues by ADC/SCC histology. (B) Clustering of Affymetrix array probes with standard deviation > 1 (11,008 or 18% of total probes) also groups tissues by ADC/SCC histology.

Quantitative proteomic analysis was performed on three lung ADC and three lung SCC tumor samples. MaxQuant reporter ion intensities for peptide expression were compared across samples, and on average we were able to identify 27.24% of the MS/MS spectra across the sample fractions or 51,001 unique peptides with posterior error probability < 0.1 and false discovery rate < 0.01. We then used Tukey’s biweight to combine these peptides into 7,241 unique proteins (see Methods for details). Unsupervised, hierarchical clustering of these proteins identified the two histologic subtypes (ADC and SCC; Fig 1A). We found 279 of 7,241 (5%) proteins to be differentially expressed between ADC and SCC (*P* < 0.05 and fold-change > 1.5 or < 0.6667). Differentially expressed proteins included p63, cytokeratin 5 and cytokeratin 6, and PKP1, which are consistent with the SCC phenotype (S2 Table) [40–43]. Enriched pathways (S3 Table) were identified using GeneGO Metacore and included cytoskeleton remodeling and cell adhesion pathways. These pathways contained p63, cytokeratins, and PKP1, again consistent with the SCC subtype.

### Comparisons of Labeled and Label-Free Proteomic Studies of NSCLC

**Fig 2:**
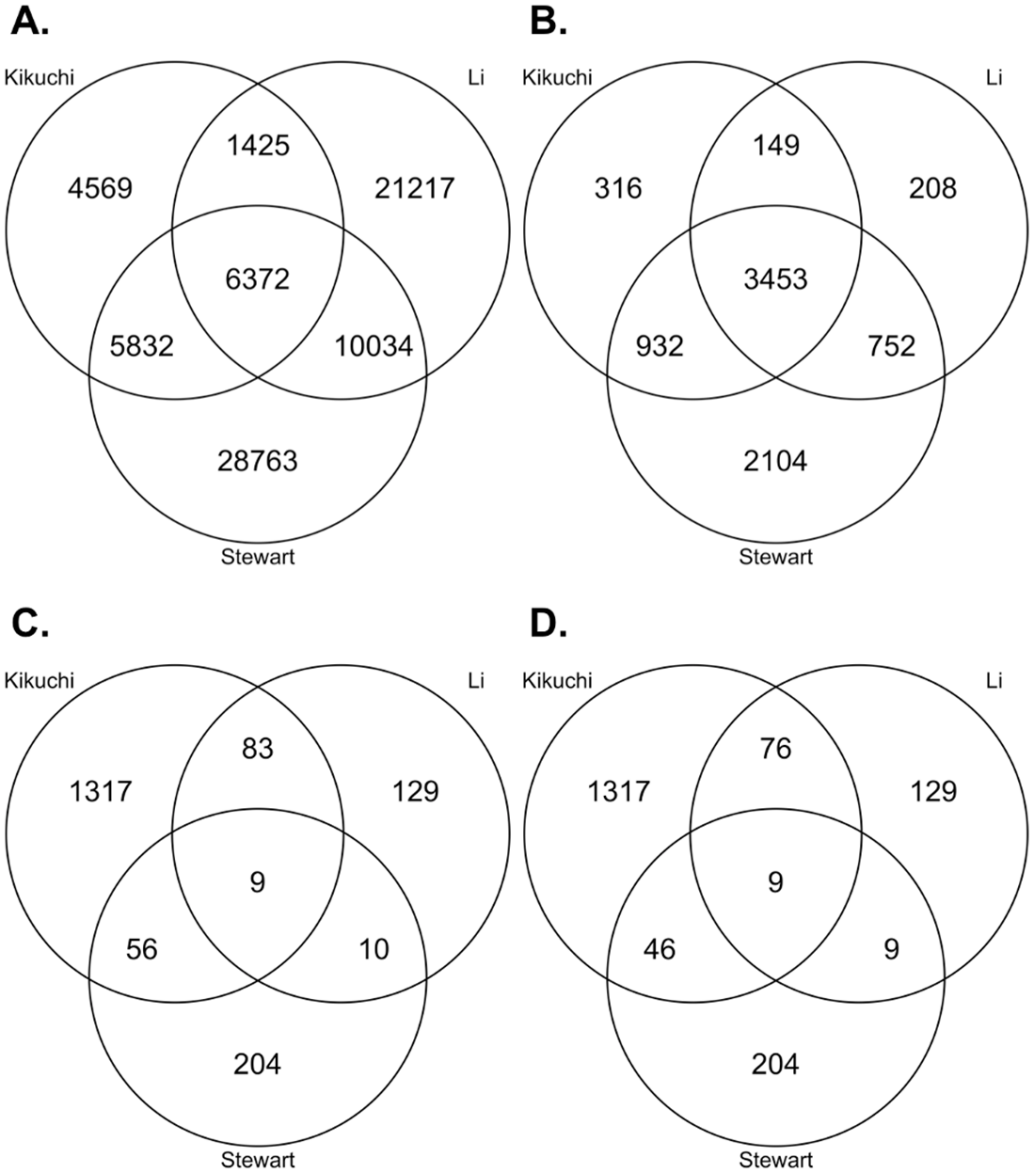
Overlap Between Proteomic Datasets. (A) Peptide overlap between proteomic datasets. (B) Protein overlap between proteomic datasets. (C) Differentially expressed protein overlap between proteomic datasets. D) Differentially expressed protein overlap between proteomic datasets, excluding proteins not sharing the same direction of change.

**Fig 3:**
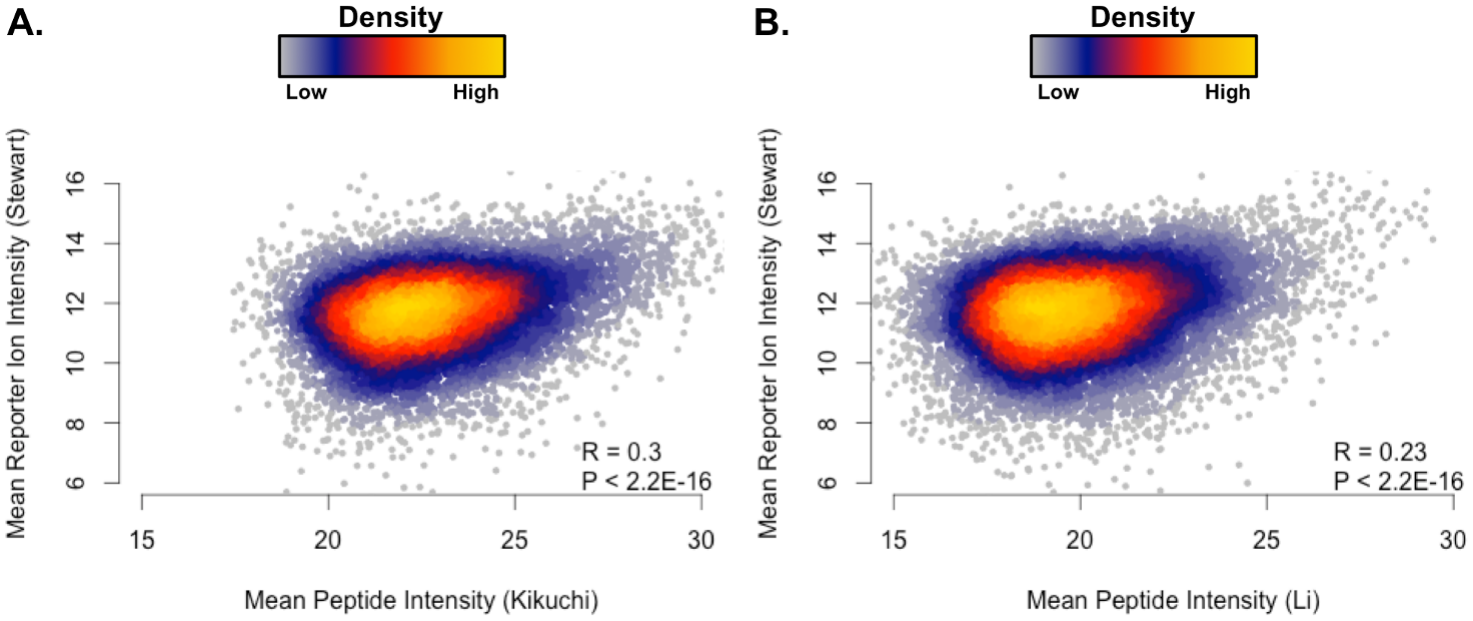
Comparison with Existing NSCLC Proteomic Datasets. Mean intensities are given in log_2_ scale. (A) The correlations between reporter ion intensities and peptide intensities from Kikuchi et al. were low (R = 0.3, *P* < 2.2E-16; ρ = 0.26, *P* < 2.2E-16). (B) As with the Kikuchi et al. data, correlations between reporter ion intensities and peptide intensities from Li et al. were also low (R = 0.23, *P* < 2.2E-16; ρ = 0.21, *P* < 2.2E-16).

We then compared our quantitative results to published proteomic studies of ADC and SCC. To reduce analytical variability in our cross-study comparison, we used MaxQuant and the same analysis pipeline to process publically available ADC and SCC lung tissue data from Kikuchi et al. and Li et al. (Table 1) [15,19]. Our dataset identified 51,001 unique peptides, the Li et al. data had 39,048 unique peptides, and the Kikuchi et al. data had 18,198 unique peptides. There were 6,372 peptides in common between the three datasets (Fig 2A). Peptides that overlapped between our results and the other studies showed low correlation (Fig 3A and 3B). Our data had 395 missing reporter ion intensities (0.1% of all peptide intensities from this dataset), the Kikuchi et al. data had 23,287 missing peptide intensity values (16% of all peptide measurement from this dataset were affected), and the Li et al. data had 92,255 missing peptide intensity values (21% of all peptide measurements from this dataset were affected). Peptide lengths (S1A Fig) between our data (mean length = 12.26) and the others (Kikuchi et al. mean length = 13.58, Li et al. mean length = 15.99) were significantly different by a Mann-Whitney/Wilcoxon rank-sum test (*P* < 0.05). This may be explained by the increased fractionation enabling additional sequencing of shorter peptides or by the increased mass and trend toward higher charge states from addition of the covalent TMT modifier.

Data from our study yielded 7,241 unique proteins (7.04 peptides per protein), the Kikuchi et al. data had 4,850 unique proteins (3.75 peptides per protein), and the Li et al. data had 4,562 unique proteins (8.56 peptides per protein). We had 1,527 proteins (21%) identified from a single peptide, the Kikuchi et al. data had 1,492 (31%), and the Li et al. data had 752 (16%) (S1B–S1D Fig). There were 3,453 proteins (48% of total identified proteome from our quantitative TMT data) in common between experiments (Fig 2B). Our data and the Kikuchi et al. data had about an order of magnitude drop (13.67 times and 14.46 times, respectively) between the number of unique identified peptides and unique identified proteins, whereas the Li et al. data showed a decrease of two orders of magnitude (102.00 times). The Kikuchi et al. data had the most differentially expressed proteins (*P* < 0.05 and fold-change > 1.5 or < 0.6667) with 1,465 (31%), the quantitative TMT data had 279 differentially expressed proteins (4%), and the Li et al. data had 231 differentially expressed proteins (5%; Fig 2C). Noticeably, the Kikuchi et al. data had 6.46 times more unique differentially expressed proteins than our data. A majority of the overlapping, differentially expressed proteins also shared the same direction of change (Fig 2D). Nine differentially expressed proteins were observed in all three datasets (Table 2), and seven of these nine differentially expressed proteins were more than two-fold higher in SCC compared to ADC. LMO7, a zinc-binding protein thought to have tumor suppressor activity in lung cancer, was the only commonly differentially expressed protein higher in ADC [44]. Both MCT1 (a lactate transporter; SLC16A1) and GLUT1 (a glucose transporter; SLC2A1) were significantly higher in SCC at the protein level (*P* < 0.05).

**Table 2:** Differentially Expressed Proteins from Quantitative TMT Shared Between Proteomic Datasets.

Protein	Peptide Count	Log_2_ Fold-Change	T-test *P* Value
KRT6C	80	2.57	0.043
*KRT6A*	*88*	*2*.*56*	*0*.*044*
*KRT6B*	*82*	*2*.*42*	*0*.*046*
PKP1	38	2.2	0.03
*MCT1*	*7*	*1*.*88*	*0*.*004*
*COL7A1*	*15*	*1*.*47*	*0*.*011*
*GLUT1*	*6*	*1*.*46*	*0*.*041*
ABCF3	11	0.73	0.013
*LMO7*	*38*	*-0*.*94*	*0*.*001*

Italics denote entries that were also differentially expressed at the gene level. Log_2_ fold-change was calculated as log_2_(SCC/ADC).

### Combining Differentially Expressed Proteins Across Studies Yields a Number of Significantly Enriched Pathways in ADC and SCC

Differentially expressed proteins from each study were simultaneously searched against GeneGO (Table 3, S4 Table). Eighty-four pathways were found enriched in SCC and 44 were enriched in ADC (*P* < 0.01). We found Wnt-related pathways enriched in the SCC subtype, consistent with previous findings [41]. Proteins related to glycolysis, including MCT1, were downregulated when Wnt signaling was inhibited in colon cancer cells [45], so the enrichment of Wnt signaling and the expression of glycolytic enzymes like MCT1 and GLUT1 may be similarly linked in SCC. We also found a glutathione metabolism pathway enriched in SCC (S4 Table). This is intriguing since there are known links between glutathione and cancer as well as drug resistance in lung cancer [46,47], and it has been shown that inhibiting MCT1 disables glutathione synthesis and glycolysis in cancer cells [48].

**Table 3:** Top Enriched Pathways from Combined Results.

Pathway	No. of Proteins	*P* Value
**Enriched in SCC (higher relative to ADC)**		
Cytoskeleton remodeling—Keratin filaments	21	2.61E-17
Cytoskeleton remodeling—Cytoskeleton remodeling	30	6.78E-14
Cytoskeleton remodeling—TGF, WNT and cytoskeletal remodeling	25	4.32E-09
Cytoskeleton remodeling—Reverse signaling by ephrin B	10	6.80E-06
LRRK2 in neurons in Parkinson's disease	12	1.80E-07
Development—TGF-beta-dependent induction of EMT via RhoA, PI3K and ILK.	14	2.20E-07
Cell adhesion—Endothelial cell contacts by junctional mechanisms	10	1.07E-06
Blood coagulation—Blood coagulation	12	1.43E-06
Cytoskeleton remodeling—Regulation of actin cytoskeleton by Rho GTPases	9	3.16E-06
Cytoskeleton remodeling—Reverse signaling by ephrin B	10	6.80E-06
**Enriched in ADC (higher relative to SCC)**		
Immune response—Antigen presentation by MHC class II	6	3.18E-07
Protein folding and maturation—Posttranslational processing of neuroendocrine peptides	8	5.57E-05
Transport—Clathrin-coated vesicle cycle	9	1.26E-04
Transport—Alpha-2 adrenergic receptor regulation of ion channels	7	2.60E-04
Cytoskeleton remodeling—Keratin filaments	6	3.86E-04
Cell adhesion—Tight junctions	6	3.86E-04
Cell adhesion—Endothelial cell contacts by junctional mechanisms	5	6.04E-04
Neurophysiological process—Dopamine D2 receptor transactivation of PDGFR in CNS	5	6.04E-04
Mitochondrial dysfunction in neurodegenerative diseases	7	1.07E-03
Cell adhesion—Gap junctions	5	1.20E-03

### Gene Expression Recapitulates Known Differences Between NSCLC Subtypes and Demonstrates Minor Protein-Gene Expression Correlation

**Fig 4:**
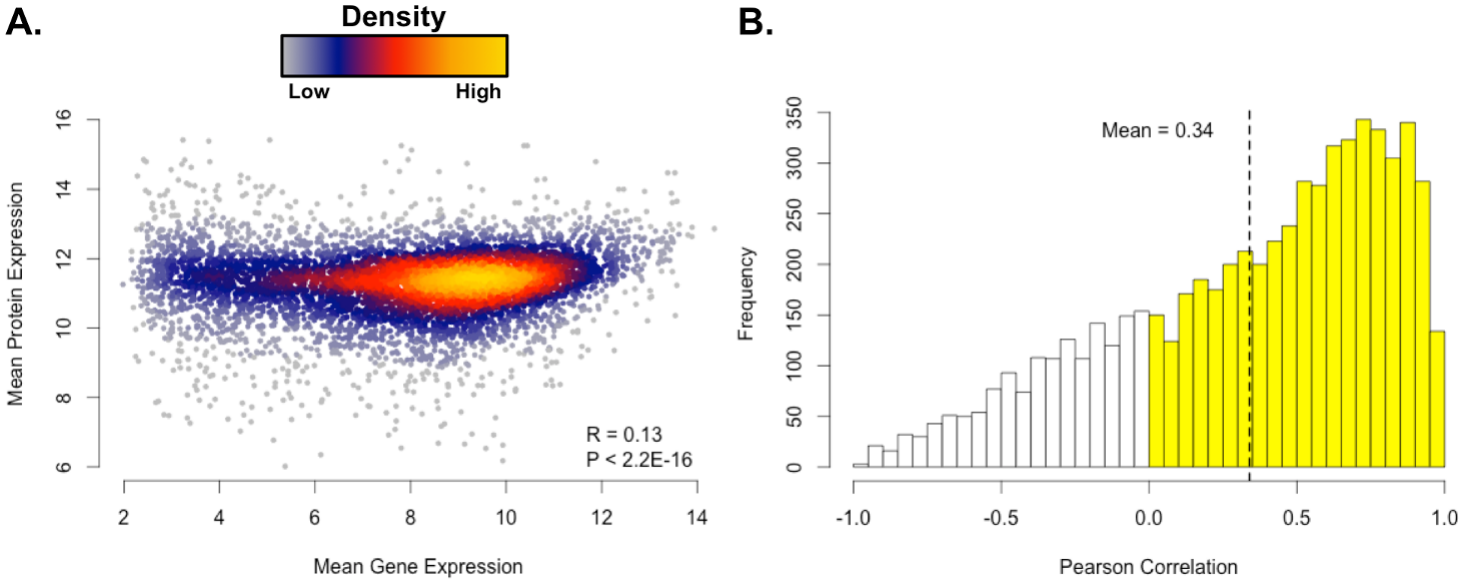
Gene and Protein Expression Show Minor Correlations Across Samples. Mean intensities are given in log_2_ scale. (A) Pairwise protein expression and corresponding gene expression across all samples. (B) Pearson correlations grouped by protein and corresponding gene. Values with Pearson R > 0 are colored yellow. The mean Pearson correlation, including all positive and negative correlations, was 0.34.

An Affymetrix microarray was used to characterize the same cohort of 3 ADC tissue and 3 SCC tissues from our quantitative proteomic study (S5 Table). We first clustered gene expression to determine whether it could recapitulate the ADC and SCC subtypes in a small sample set (Fig 1B). We then identified 6,373 genes with matching protein expression for further analysis. The pairwise correlation between all protein expressions and gene expressions was low (R = 0.13, *P* < 2.2E-16; ρ = 0.16, *P* < 2.2E-16) (Fig 4A), but correlations grouped by protein and corresponding gene were higher by Pearson’s method (mean R = 0.34, Fig 4B) and Spearman’s method (mean ρ = 0.31). These results are comparable with the recent CPTAC colon cancer proteogenomic study (ρ = 0.23) as well as results from Wilhelm et al. (ρ = 0.31 to 0.56) [3,49]. We found 980 gene/protein observations to be significantly correlated by Pearson’s method (*P* < 0.05 and R > 0.5; Table 4) and 582 by Spearman’s method (*P* < 0.05 and ρ > 0.5). Of the 6,373 matched genes, 629 were differentially expressed (*P* < 0.05) and included genes consistent with the ADC and SCC subtypes (e.g., p63 expression, cytokeratin expression). Similarly, 493 of the 6,373 matched proteins were significant (*P* < 0.05). Looking at the intersection of the datasets, 119 were differentially expressed at both the gene and protein levels. As expected, the mean correlation between these 119 significant gene and protein pairs was higher (R = 0.79; ρ = 0.76), and 113 pairs (95%) changed in the same direction between ADC and SCC. Six of the nine commonly differentially expressed proteins in Table 2 were also differentially expressed at the gene expression level. This included both the MCT1 gene expression that was 9.75 times higher in SCC than in ADC (*P* = 0.06; S5 Table) and the GLUT1 gene expression that was 4.78 times higher in SCC compared to ADC (*P* = 0.02; S5 Table).

**Table 4:** Gene and Protein Expression Agreement.

No. matched	6373
No. significantly correlated	980
No. differentially expressed proteins	493
No. differentially expressed genes	629
No. gene/protein overlap	119
Mean Pearson Correlation	0.79
No. Fold-change agreement	113

### Protein Expression, Gene Expression, and Survival Analysis Suggest MCT1 and GLUT1 May Be Prognostic Markers in ADC but not in SCC

**Fig 5:**
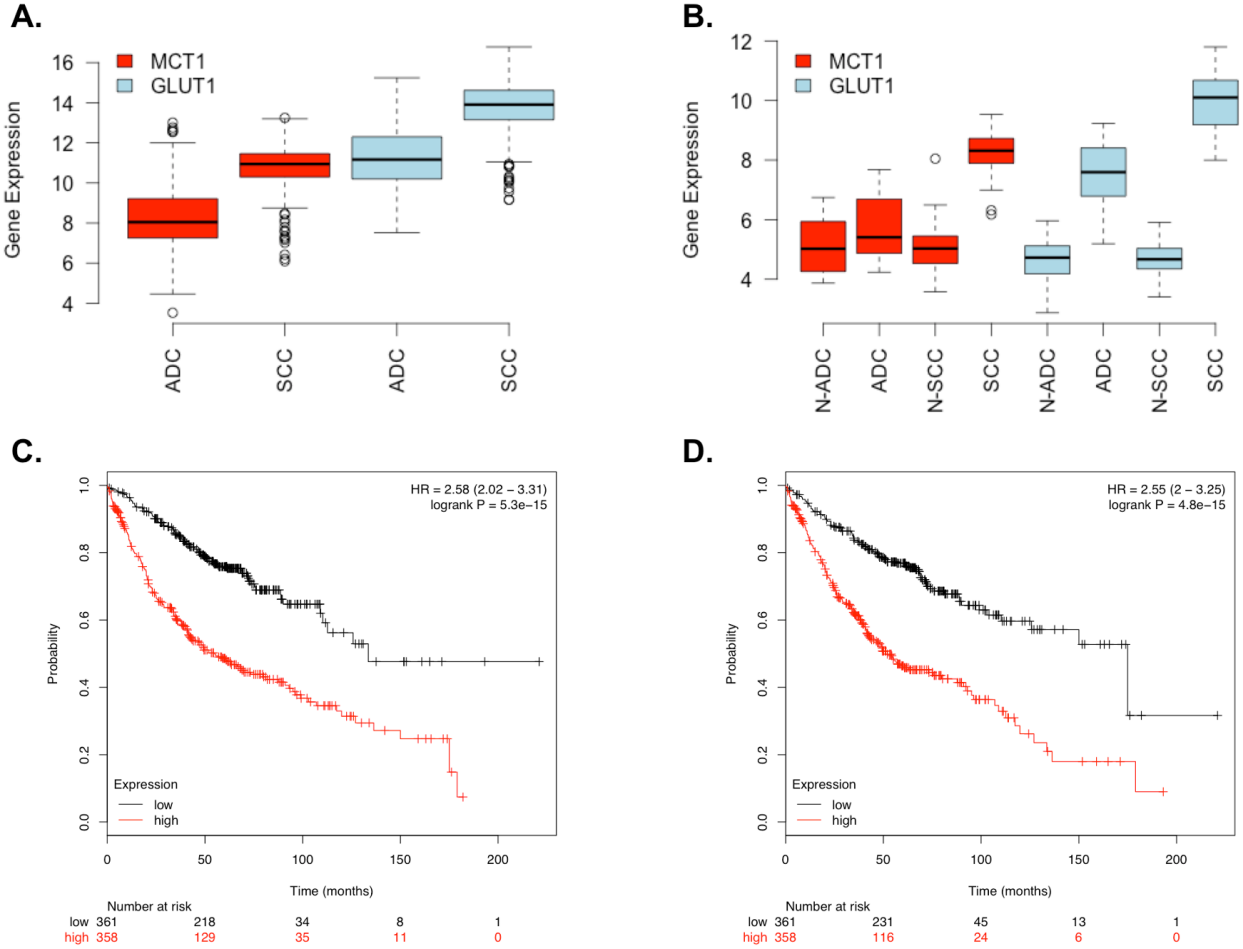
Expression and Survival Analysis of MCT1 and GLUT1 in NSCLC Datasets. Gene expression is given in log_2_ scale. (A) Expression of MCT1 and GLUT1 in TCGA data (ADC = 490, SCC = 491). (B) Expression of MCT1 and GLUT1 ADC, SCC, and matched normal samples (N-ADC and N-SCC, respectively) extracted from Sanchez-Palencia et al.[55] (C) Survival analysis of ADC patients expressing low and high levels of MCT1 (median cutoff). (D) Survival analysis of ADC patients expressing low and high levels of GLUT1 (median cutoff).

We chose to further investigate the implications of the MCT1 and GLUT1 findings since cancer cells rely on aerobic glycolysis and produce excess lactate via the Warburg effect [50]. MCT1 is expressed in most tissues at low levels [51], and MCT1 is known to be higher in squamous cell lung cancer and metastatic lung cancer [41,48,52–56]. The primary studies by Kikuchi et al. and Li et al. both did not identify MCT1 in normal samples but did in SCC samples. Kikuchi et al. did not identify MCT1 in ADC samples; however, Li et al. did identify MCT1 in one ADC sample. An analysis of three microarray datasets by Shi et al. found MCT1 consistently upregulated in SCC [41]. Schuurbiers et al. observed an increase of MCT1 and GLUT1 in SCC over ADC via immunofluorescent staining and qPCR [56]. At the RNA expression level, both MCT1 and GLUT1 were significantly higher in SCC than in ADC in TCGA lung cancer data (ADC = 490, SCC = 491, *P* < 2.2E-16; Fig 5A). We found similar findings in the Sanchez-Palencia et al. GEO dataset (GSE18842; ADC = 12, SCC = 31): MCT1 and GLUT1 gene expression were significantly higher in SCC than in ADC (5.71 times higher/*P* = 6.06E-6 and 5.96 times higher/*P* = 3.21E-6, respectively; Fig 5B) [55]. Furthermore, MCT1 gene expression in ADC tumors and compared to matched normal tissues was not significantly different (*P* = 0.18), but GLUT1 gene expression was 7.17 times higher in ADC tumors than in matched normal samples (*P* = 1.40E-06; Fig 5B). MCT1 and GLUT1 gene expression were both significantly higher in SCC tumors compared to matched normal tissues (9.16 times higher/*P* < 2.2E-16 and 41.08 times higher/*P* < 2.2E-16, respectively; Fig 5B).

Elevated MCT1 is reported to be a hallmark of MYC-driven malignancies [48]. We did not observe MYC, MYCL, or MYCN in our quantitative TMT results, the Kikuchi et al. results, or the Li et al. results; therefore, we tested the relationships between MYC, MYCL, MYCN, and MCT1 in the aforementioned TCGA lung cancer dataset to further investigate. MYC was 2.29 times higher (*P* < 2.2E-16), MYCL was 2.13 times higher (*P* < 2.2E-16), and MYCN was 1.31 times higher (*P* < 2.2E-16) in SCC compared with ADC. However, the correlation between MYC and MCT1 was about the same in both ADC (R = 0.28, *P* = 3.31E-10; ρ = 0.28, *P* = 9.46E-10) and SCC (R = 0.27, *P* = 1.05E-9; ρ = 0.23, *P* = 2.12E-07). Correlation between MYCL and MCT1 was negligible in ADC (R = -0.06, *P* = 0.21; ρ = -0.13, *P* = 4.84E-3) and in SCC (R = -0.06, *P* = 0.20; ρ = -0.04, *P* = 0.37). Correlation between MYCN and MCT1 was also negligible in both ADC (R = -2.94E-3, *P* = 0.94; ρ = 3.29E-3, *P* = 0.94) and SCC (R = -5.7E-3, *P* = 0.90; ρ = -0.06, *P* = 0.19). It is possible that elevated MYC expression in SCC tissues could be causing downstream elevation of MCT1, but there is not clear numerical evidence to support this.

Interestingly, Eilertsen et al. found MCT1 to be an independent prognostic marker for survival in NSCLC patients [53]. Furthermore, this same study shows that high expression of both MCT1 and GLUT1 has a negative impact on survival in NSCLC patients. Eilertsen’s patient cohort included both ADC and SCC, and, although a majority of patients had the SCC subtype, it is unclear whether this finding would hold for just one subtype. To address this, we performed survival analysis to further investigate the subtype-specific role of MCT1 and GLUT1 in NSCLC. Intriguingly, high expression of MCT1 was not associated with poor survival in SCC patients (*P* = 0.9, HR = 0.98; CI = 0.78–1.25; n = 525; S2 Fig) but was in ADC patients (*p* = 5.3E-15, HR = 2.58, CI = 2.02–3.31, n = 719; Fig 5C). Similarly, high expression of GLUT1 was not associated with poor survival in SCC patients (*P* = 0.063, HR = 1.25, CI = 0.99–1.59; n = 525; S3 Fig) but was in ADC patients (*P* = 4.8E-15, HR = 2.55, CI = 2–3.25, n = 719; Fig 5D). Our findings suggest that MCT1 and GLUT1 are both commonly overexpressed at the gene and protein levels in SCC and thus not prognostic. However, they may serve as potential histological markers or targets for this subtype since they are overexpressed in SCC tumors compared to normal tissue. On the other hand, there appears to be a subpopulation of ADC samples with overexpression of MCT1 and GLUT1, and we suggest that MCT1 and GLUT1 could serve as prognostic markers in the ADC subtype since their overexpression is associated with poor survival. MCT1 and GLUT1 were associated with poor survival in ADC (MCT1 *P* = 0.0016; GLUT1 *P* = 0.0005) even after accounting for stage, gender, and smoking history in multivariate survival analyses (S6 Table). We note that an MCT1 inhibitor is being evaluated for its efficacy in small cell lung cancer in the United Kingdom [57].

## Discussion

ADC and SCC are very heterogeneous diseases, and this statement is emphasized by the existence of several subtypes within ADC and SCC [58,59]. Making sense of the variations between ADC and SCC can be a difficult task, but alterations in the genomes of these cancers ultimately get integrated and produce a cancer proteome that can be analyzed using modern proteomic tools. However, it is already known that the agreement between ADC gene expression and protein expression is weak, underscoring the need for both genomic and proteomic tools in order to identify differences and ultimately biomarkers and drug targets in NSCLC [60,61]. Here, we undertook a pilot proteogenomic to understand how TMT would perform in our hands experimentally as well as whether special considerations would arise while analyzing TMT-derived data. Protein expression from our experiments shared some overlap with the gene expression, and both protein and gene expression were able to group tissues by ADC and SCC histology (Fig 1). Our gene-protein correlation was similar to previously published results [3,49], and our findings were better than a previous study that had shown poor agreement of gene and protein expression in ADC (ρ = −0.025) [60].

We then compared our results to previously published studies. We reduced variability in our analysis by processing all raw discovery proteomics data through MaxQuant. However, experimental differences, including patient sample differences, were sources of uncontrollable variability. The number of samples and sample fractions, as well as the LC-MS gradients and MS analysis time, certainly influenced the number of identified peptides from each study. For example, we divided our samples into 50 fractions in order to minimize any co-isolation events during precursor ion isolation prior to MS^2^ (Table 1), and we believe that this large fractionation directly led to the large number of uniquely identified peptides observed in our study (Fig 2). Li et al. utilized a 5-h run time without any fractionation, and Kikuchi et al. used 20 fractions and utilized a 90-min run time. The Kikuchi et al. data did have fewer identified peptides, but this may be because their samples (ADC = 4 and SCC = 4) were the same pools of samples, one for ADC and one for SCC, which were run four separate times. However, this finding is balanced by the fact that the Kikuchi et al. data had the best protein representation (Table 1).

Despite the differences in the content of the discovery proteomics experiments, there was a benefit in having these previous results to compare and analyze with our own. We were able to identify nine differentially expressed proteins in common with the other datasets that also had the same increase or decrease in expression relative to ADC and SCC (Fig 2C; Table 2), and we were able to identify a number of pathways by simultaneously searching all differentially expressed proteins from each study against GeneGO (Table 3, S4 Table). Of the nine common differentially expressed proteins, four were known to be highly expressed in SCC: three isoforms of keratin 6 as well as PKP1 [40–43]. LMO7 was the only commonly differentially expressed protein higher in ADC. CO7A1, a collagen, and ABCF3, an ATP-binding cassette family member, were also consistently differentially expressed. However, there does not appear to be an obvious link between these two proteins and the ADC or SCC subtypes, and we note that the ABCF3 expression in our dataset was only modestly higher in our TMT SCC results (0.73 log_2_ fold-change).

The most interesting of the common differentially expressed proteins were the lactate transporter MCT1 and glucose transporter GLUT1. Because cancer cells rely on aerobic glycolysis and produce excess lactate via the Warburg effect, we wished to understand how these transporters behaved in a larger cohort as well as if they would be differentially expressed compared to normal tissues. Data analysis of publically available datasets (Fig 5A and 5B) showed that MCT1 and GLUT1 were constitutively overexpressed in SCC and therefore may make promising drug targets or histological markers for this subtype. Survival analysis of publically available datasets (Fig 5C and 5D) showed that higher expression of MCT1 and GLUT1 are associated with poor survival in ADC patients and therefore may make promising prognostic markers. Our findings suggest a larger role for MCT1 and GLUT1 in ADC and SCC than what is currently understood, and further work should be undertaken to elucidate their function in these NSCLC subtypes.

## Conclusions

These results suggest that our approach using quantitative TMT is a valid approach for future and more expansive proteogenomic studies of cancer. Further, recent studies have found that TMTs were more sensitive than iTRAQ [62]. Additional improvements in throughput can be gained by reducing the number of fractions since other studies have reported large inventories with less fractionation and instrument time [3,63,64]. Finally, switching from microarrays to next generation sequencing technology (e.g., RNA sequencing) will enable the generation of personalized peptide libraries that are likely to increase proteome coverage through variant detection [65]. Improvements in genome and proteome technology, when coupled with high quality cohorts with clinical data and well annotated tumor tissues, are likely to enable new discoveries on proteins and pathways important in cancer.

## Supporting Information

S1 Fig Peptide Lengths and Peptides per Protein. (A) Peptide lengths differ between each dataset. (B–D) The number of peptides identified per protein in each of the studies. Samples with > 15 peptides per protein were excluded for plot clarity.
(TIF)

S2 Fig SCC MCT1 Survival. Survival analysis of SCC patients expressing low and high levels of MCT1 (median cutoff).
(TIF)

S3 Fig SCC GLUT1 Survival. Survival analysis of SCC patients expressing low and high levels of GLUT1 (median cutoff).
(TIF)

S1 Table Characteristics of Patient Tissue Samples. (XLSX)

S2 Table Quantitative TMT Results. (XLSX)

S3 Table Pathway Enrichment from Quantitative TMT Results. (XLSX)

S4 Table Pathway Enrichment from Combined Proteomics Results. (XLSX)

S5 Table Gene Expression Results. (XLSX)

S6 Table Multivariate survival analyses of MCT1 and GLUT1 in ADC. (XLSX)

## References

[1] Mann M, Kulak NA, Nagaraj N, Cox J. The Coming Age of Complete, Accurate, and Ubiquitous Proteomes. Mol Cell. 2013;49: 583–590. 10.1016/j.molcel.2013.01.029 23438854

[2] Alfaro JA, Sinha A, Kislinger T, Boutros PC. Onco-proteogenomics: cancer proteomics joins forces with genomics. Nat Methods. 2014;11: 1107–1113. 10.1038/nmeth.3138 25357240

[3] Zhang B, Wang J, Wang X, Zhu J, Liu Q, Shi Z, et al Proteogenomic characterization of human colon and rectal cancer. Nature. 2014;513: 382–387. 10.1038/nature13438 25043054 PMC4249766

[4] Nesvizhskii AI. Proteogenomics: concepts, applications and computational strategies. Nat Methods. 2014;11: 1114–1125. 10.1038/nmeth.3144 25357241 PMC4392723

[5] Sanders WS, Wang N, Bridges SM, Malone BM, Dandass YS, McCarthy FM, et al The Proteogenomic Mapping Tool. BMC Bioinformatics. 2011;12: 115 10.1186/1471-2105-12-115 21513508 PMC3107813

[6] Jagtap PD, Johnson JE, Onsongo G, Sadler FW, Murray K, Wang Y, et al Flexible and Accessible Workflows for Improved Proteogenomic Analysis Using the Galaxy Framework. J Proteome Res. 2014;13: 5898–5908. 10.1021/pr500812t 25301683 PMC4261978

[7] Tovchigrechko A, Venepally P, Payne SH. PGP: parallel prokaryotic proteogenomics pipeline for MPI clusters, high-throughput batch clusters and multicore workstations. Bioinformatics. 2014; btu051. 10.1093/bioinformatics/btu051 PMC4016709 24470574

[8] Woo S, Cha SW, Merrihew G, He Y, Castellana N, Guest C, et al Proteogenomic Database Construction Driven from Large Scale RNA-seq Data. J Proteome Res. 2014;13: 21–28. 10.1021/pr400294c 23802565 PMC4034692

[9] Boekel J, Chilton JM, Cooke IR, Horvatovich PL, Jagtap PD, Käll L, et al Multi-omic data analysis using Galaxy. Nat Biotechnol. 2015;33: 137–139. 10.1038/nbt.3134 25658277

[10] McAlister GC, Huttlin EL, Haas W, Ting L, Jedrychowski MP, Rogers JC, et al Increasing the multiplexing capacity of TMT using reporter ion isotopologues with isobaric masses. Anal Chem. 2012;84: 7469–7478. 10.1021/ac301572t 22880955 PMC3715028

[11] Pastor MD, Nogal A, Molina-Pinelo S, Carnero A, Paz-Ares L. Proteomic biomarkers in lung cancer. Clin Transl Oncol. 2013;15: 671–682. 10.1007/s12094-013-1034-0 23606351

[12] Lehtiö J, De Petris L. Lung cancer proteomics, clinical and technological considerations. J Proteomics. 2010;73: 1851–1863. 10.1016/j.jprot.2010.05.015 20685322

[13] Tanvetyanon T, Creelan BC, Chiappori AA. Current clinical application of genomic and proteomic profiling in non-small-cell lung cancer. Cancer Control J Moffitt Cancer Cent. 2014;21: 32–39. 10.1177/107327481402100105 24357739

[14] Wei Y, Tong J, Taylor P, Strumpf D, Ignatchenko V, Pham N-A, et al Primary tumor xenografts of human lung adeno and squamous cell carcinoma express distinct proteomic signatures. J Proteome Res. 2011;10: 161–174. 10.1021/pr100491e 20815376

[15] Kikuchi T, Hassanein M, Amann JM, Liu Q, Slebos RJC, Rahman SMJ, et al In-depth Proteomic Analysis of Nonsmall Cell Lung Cancer to Discover Molecular Targets and Candidate Biomarkers. Mol Cell Proteomics. 2012;11: 916–932. 10.1074/mcp.M111.015370 22761400 PMC3494148

[16] Zhang W, Wei Y, Ignatchenko V, Li L, Sakashita S, Pham N-A, et al Proteomic profiles of human lung adeno and squamous cell carcinoma using super-SILAC and label-free quantification approaches. PROTEOMICS. 2014;14: 795–803. 10.1002/pmic.201300382 24453208

[17] Linxweiler J, Kollipara L, Zahedi RP, Lampel P, Zimmermann R, Greiner M. Proteomic insights into non-small cell lung cancer: New ideas for cancer diagnosis and therapy from a functional viewpoint. EuPA Open Proteomics. 2014;4: 25–39. 10.1016/j.euprot.2014.05.004

[18] Sun H, Xing X, Li J, Zhou F, Chen Y, He Y, et al Identification of gene fusions from human lung cancer mass spectrometry data. BMC Genomics. 2013;14: S5 10.1186/1471-2164-14-S8-S5 PMC4042237 24564548

[19] Li L, Wei Y, To C, Zhu C-Q, Tong J, Pham N-A, et al Integrated Omic analysis of lung cancer reveals metabolism proteome signatures with prognostic impact. Nat Commun. 2014;5 10.1038/ncomms6469 25429762

[20] Cox J, Mann M. MaxQuant enables high peptide identification rates, individualized p.p.b.-range mass accuracies and proteome-wide protein quantification. Nat Biotechnol. 2008;26: 1367–1372. 10.1038/nbt.1511 19029910

[21] Manza LL, Stamer SL, Ham A-JL, Codreanu SG, Liebler DC. Sample preparation and digestion for proteomic analyses using spin filters. Proteomics. 2005;5: 1742–1745. 10.1002/pmic.200401063 15761957

[22] Wiśniewski JR, Zougman A, Nagaraj N, Mann M. Universal sample preparation method for proteome analysis. Nat Methods. 2009;6: 359–362. 10.1038/nmeth.1322 19377485

[23] Bennett KL, Funk M, Tschernutter M, Breitwieser FP, Planyavsky M, Mohien CU, et al Proteomic analysis of human cataract aqueous humour: Comparison of one-dimensional gel LCMS with two-dimensional LCMS of unlabelled and iTRAQ^®^-labelled specimens. J Proteomics. 2011;74: 151–166. 10.1016/j.jprot.2010.10.002 20940065

[24] Olsen JV, de Godoy LMF, Li G, Macek B, Mortensen P, Pesch R, et al Parts per million mass accuracy on an Orbitrap mass spectrometer via lock mass injection into a C-trap. Mol Cell Proteomics MCP. 2005;4: 2010–2021. 10.1074/mcp.T500030-MCP200 16249172

[25] Vizcaíno JA, Deutsch EW, Wang R, Csordas A, Reisinger F, Ríos D, et al ProteomeXchange provides globally coordinated proteomics data submission and dissemination. Nat Biotechnol. 2014;32: 223–226. 10.1038/nbt.2839 24727771 PMC3986813

[26] Cox J, Neuhauser N, Michalski A, Scheltema RA, Olsen JV, Mann M. Andromeda: a peptide search engine integrated into the MaxQuant environment. J Proteome Res. 2011;10: 1794–1805. 10.1021/pr101065j 21254760

[27] Wenig P, Odermatt J. OpenChrom: a cross-platform open source software for the mass spectrometric analysis of chromatographic data. BMC Bioinformatics. 2010;11: 405 10.1186/1471-2105-11-405 20673335 PMC2920884

[28] Eschrich SA, Hoerter AM. Libaffy: software for processing Affymetrix(R) GeneChip(R) data. Bioinformatics. 2007;23: 1562–1564. 10.1093/bioinformatics/btm127 17463032

[29] Eschrich SA, Hoerter AM, Bloom GC, Fenstermacher DA. Tissue-specific RMA models to incrementally normalize Affymetrix GeneChip data. 30th Annual International Conference of the IEEE Engineering in Medicine and Biology Society, 2008 EMBS 2008. 2008 pp. 2419–2422. 10.1109/IEMBS.2008.4649687 19163190

[30] Welsh EA, Eschrich SA, Berglund AE, Fenstermacher DA. Iterative rank-order normalization of gene expression microarray data. BMC Bioinformatics. 2013;14: 153 10.1186/1471-2105-14-153 23647742 PMC3651355

[31] R Core Team. R: A language and environment for statistical computing. Vienna, Austria: R Foundation for Statistical Computing; 2014.

[32] RStudio: Integrated development environment for R. Boston, MA: RStudio; 2014.

[33] Nesvizhskii AI, Aebersold R. Interpretation of Shotgun Proteomic Data The Protein Inference Problem. Mol Cell Proteomics. 2005;4: 1419–1440. 10.1074/mcp.R500012-MCP200 16009968

[34] Cox J, Mann M. 1D and 2D annotation enrichment: a statistical method integrating quantitative proteomics with complementary high-throughput data. BMC Bioinformatics. 2012;13: S12 10.1186/1471-2105-13-S16-S12 PMC3489530 23176165

[35] Karpievitch YV, Dabney AR, Smith RD. Normalization and missing value imputation for label-free LC-MS analysis. BMC Bioinformatics. 2012;13: S5 10.1186/1471-2105-13-S16-S5 PMC3489534 23176322

[36] Hoaglin DC, Mosteller F, Tukey JW. Understanding robust and exploratory data analysis. Wiley; 2000.

[37] Gentleman RC, Carey VJ, Bates DM, Bolstad B, Dettling M, Dudoit S, et al Bioconductor: open software development for computational biology and bioinformatics. Genome Biol. 2004;5: R80 10.1186/gb-2004-5-10-r80 15461798 PMC545600

[38] Gautier L, Cope L, Bolstad BM, Irizarry RA. affy—analysis of Affymetrix GeneChip data at the probe level. Bioinformatics. 2004;20: 307–315. 10.1093/bioinformatics/btg405 14960456

[39] Győrffy B, Surowiak P, Budczies J, Lánczky A. Online survival analysis software to assess the prognostic value of biomarkers using transcriptomic data in non-small-cell lung cancer. PloS One. 2013;8: e82241 10.1371/journal.pone.0082241 24367507 PMC3867325

[40] Terry J, Leung S, Laskin J, Leslie KO, Gown AM, Ionescu DN. Optimal immunohistochemical markers for distinguishing lung adenocarcinomas from squamous cell carcinomas in small tumor samples. Am J Surg Pathol. 2010;34: 1805–1811. 10.1097/PAS.0b013e3181f7dae3 21107086

[41] Shi I, Hashemi Sadraei N, Duan Z-H, Shi T. Aberrant Signaling Pathways in Squamous Cell Lung Carcinoma. Cancer Inform. 2011;10: 273–285. 10.4137/CIN.S8283 22174565 PMC3236010

[42] Rekhtman N, Ang DC, Sima CS, Travis WD, Moreira AL. Immunohistochemical algorithm for differentiation of lung adenocarcinoma and squamous cell carcinoma based on large series of whole-tissue sections with validation in small specimens. Mod Pathol Off J U S Can Acad Pathol Inc. 2011;24: 1348–1359. 10.1038/modpathol.2011.92 21623384

[43] Gómez-Morales M, Cámara-Pulido M, Miranda-León MT, Sánchez-Palencia A, Boyero L, Gómez-Capilla JA, et al Differential immunohistochemical localization of desmosomal plaque-related proteins in non-small-cell lung cancer. Histopathology. 2013;63: 103–113. 10.1111/his.12126 23711109

[44] Tanaka-Okamoto M, Hori K, Ishizaki H, Hosoi A, Itoh Y, Wei M, et al Increased susceptibility to spontaneous lung cancer in mice lacking LIM-domain only 7. Cancer Sci. 2009;100: 608–616. 10.1111/j.1349-7006.2009.01091.x 19215226 PMC11159906

[45] Pate KT, Stringari C, Sprowl‐Tanio S, Wang K, TeSlaa T, Hoverter NP, et al Wnt signaling directs a metabolic program of glycolysis and angiogenesis in colon cancer. EMBO J. 2014;33: 1454–1473. 10.15252/embj.201488598 24825347 PMC4194089

[46] Balendiran GK, Dabur R, Fraser D. The role of glutathione in cancer. Cell Biochem Funct. 2004;22: 343–352. 10.1002/cbf.1149 15386533

[47] Yang P, Ebbert JO, Sun Z, Weinshilboum RM. Role of the Glutathione Metabolic Pathway in Lung Cancer Treatment and Prognosis: A Review. J Clin Oncol. 2006;24: 1761–1769. 10.1200/JCO.2005.02.7110 16603718

[48] Doherty JR, Yang C, Scott KEN, Cameron MD, Fallahi M, Li W, et al Blocking Lactate Export by Inhibiting the Myc Target MCT1 Disables Glycolysis and Glutathione Synthesis. Cancer Res. 2014;74: 908–920. 10.1158/0008-5472.CAN-13-2034 24285728 PMC3946415

[49] Wilhelm M, Schlegl J, Hahne H, Gholami AM, Lieberenz M, Savitski MM, et al Mass-spectrometry-based draft of the human proteome. Nature. 2014;509: 582–587. 10.1038/nature13319 24870543

[50] Vander Heiden MG, Cantley LC, Thompson CB. Understanding the Warburg Effect: The Metabolic Requirements of Cell Proliferation. Science. 2009;324: 1029–1033. 10.1126/science.1160809 19460998 PMC2849637

[51] Doherty JR, Cleveland JL. Targeting lactate metabolism for cancer therapeutics. J Clin Invest. 2013;123: 3685–3692. 10.1172/JCI69741 23999443 PMC3754272

[52] Kuner R, Muley T, Meister M, Ruschhaupt M, Buness A, Xu EC, et al Global gene expression analysis reveals specific patterns of cell junctions in non-small cell lung cancer subtypes. Lung Cancer. 2009;63: 32–38. 10.1016/j.lungcan.2008.03.033 18486272

[53] Eilertsen M, Andersen S, Al-Saad S, Kiselev Y, Donnem T, Stenvold H, et al Monocarboxylate Transporters 1–4 in NSCLC: MCT1 Is an Independent Prognostic Marker for Survival. PLoS ONE. 2014;9: e105038 10.1371/journal.pone.0105038 25225794 PMC4165596

[54] Lee G-H, Kim D-S, Chung MJ, Chae S-W, Kim H-R, Chae H-J. Lysyl oxidase-like-1 enhances lung metastasis when lactate accumulation and monocarboxylate transporter expression are involved. Oncol Lett. 2011;2: 831–838. 10.3892/ol.2011.353 22866136 PMC3408044

[55] Sanchez-Palencia A, Gomez-Morales M, Gomez-Capilla JA, Pedraza V, Boyero L, Rosell R, et al Gene expression profiling reveals novel biomarkers in nonsmall cell lung cancer. Int J Cancer J Int Cancer. 2011;129: 355–364. 10.1002/ijc.25704 20878980

[56] Schuurbiers OCJ, Meijer TWH, Kaanders JHAM, Looijen-Salamon MG, de Geus-Oei L-F, van der Drift MA, et al Glucose Metabolism in NSCLC Is Histology-Specific and Diverges the Prognostic Potential of 18FDG-PET for Adenocarcinoma and Squamous Cell Carcinoma: J Thorac Oncol. 2014;9: 1485–1493. 10.1097/JTO.0000000000000286 25170642

[57] Polański R, Hodgkinson CL, Fusi A, Nonaka D, Priest L, Kelly P, et al Activity of the monocarboxylate transporter 1 inhibitor AZD3965 in small cell lung cancer. Clin Cancer Res Off J Am Assoc Cancer Res. 2014;20: 926–937. 10.1158/1078-0432.CCR-13-2270 PMC3929348 24277449

[58] The Cancer Genome Atlas Research Network. Comprehensive genomic characterization of squamous cell lung cancers. Nature. 2012;489: 519–525. 10.1038/nature11404 22960745 PMC3466113

[59] The Cancer Genome Atlas Research Network. Comprehensive molecular profiling of lung adenocarcinoma. Nature. 2014;511: 543–550. 10.1038/nature13385 25079552 PMC4231481

[60] Chen G, Gharib TG, Huang C-C, Taylor JMG, Misek DE, Kardia SLR, et al Discordant protein and mRNA expression in lung adenocarcinomas. Mol Cell Proteomics MCP. 2002;1: 304–313. 12096112 10.1074/mcp.m200008-mcp200

[61] Boja ES, Rodriguez H. Proteogenomic convergence for understanding cancer pathways and networks. Clin Proteomics. 2014;11: 22 10.1186/1559-0275-11-22 24994965 PMC4067069

[62] Sandberg A, Branca RMM, Lehtiö J, Forshed J. Quantitative accuracy in mass spectrometry based proteomics of complex samples: the impact of labeling and precursor interference. J Proteomics. 2014;96: 133–144. 10.1016/j.jprot.2013.10.035 24211767

[63] Moghaddas Gholami A, Hahne H, Wu Z, Auer FJ, Meng C, Wilhelm M, et al Global proteome analysis of the NCI-60 cell line panel. Cell Rep. 2013;4: 609–620. 10.1016/j.celrep.2013.07.018 23933261

[64] Kim M-S, Pinto SM, Getnet D, Nirujogi RS, Manda SS, Chaerkady R, et al A draft map of the human proteome. Nature. 2014;509: 575–581. 10.1038/nature13302 24870542 PMC4403737

[65] Li J, Su Z, Ma ZQ, Slebos RJ, Halvey P, Tabb DL, et al A bioinformatics workflow for variant peptide detection in shotgun proteomics. Mol Cell Proteomics. 2011;10(5):M110 006536 10.1074/mcp.M110.006536 21389108 PMC3098595

