# Supplementary figures and images for: A Pilot Proteogenomic Study with Data Integration Identifies MCT1 and GLUT1 as Prognostic Markers in Lung Adenocarcinoma
Source: PLoS One. 2015 Nov 5;10(11):e0142162. doi: 10.1371/journal.pone.0142162 (PMC4634858; doi:10.1371/journal.pone.0142162)

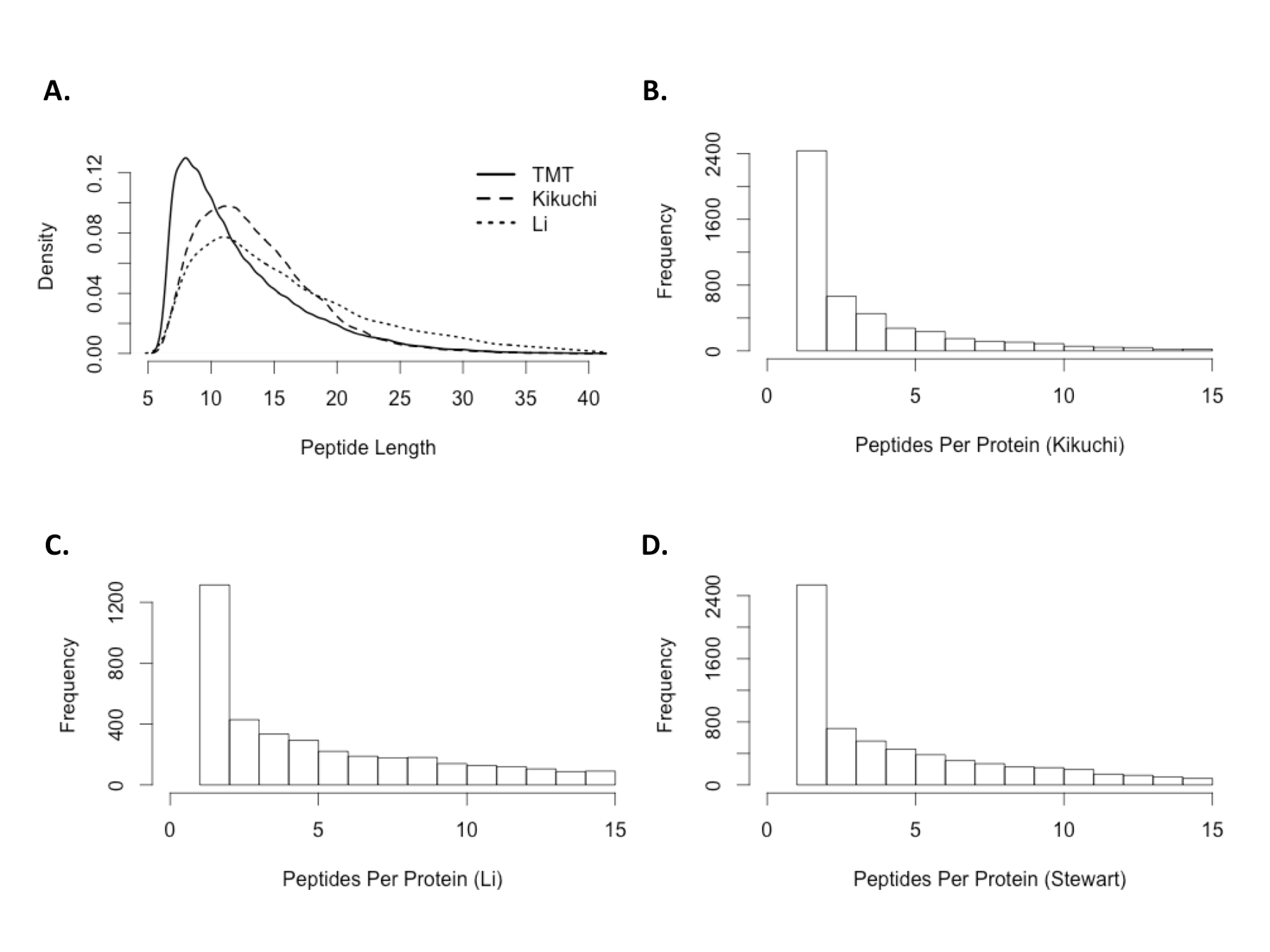

Supplement: S1 Fig — (A) Peptide lengths differ between each dataset. (B–D) The number of peptides identified per protein in each of the studies. Samples with > 15 peptides per protein were excluded for plot clarity. (TIF) [file pone.0142162.s001.tif]

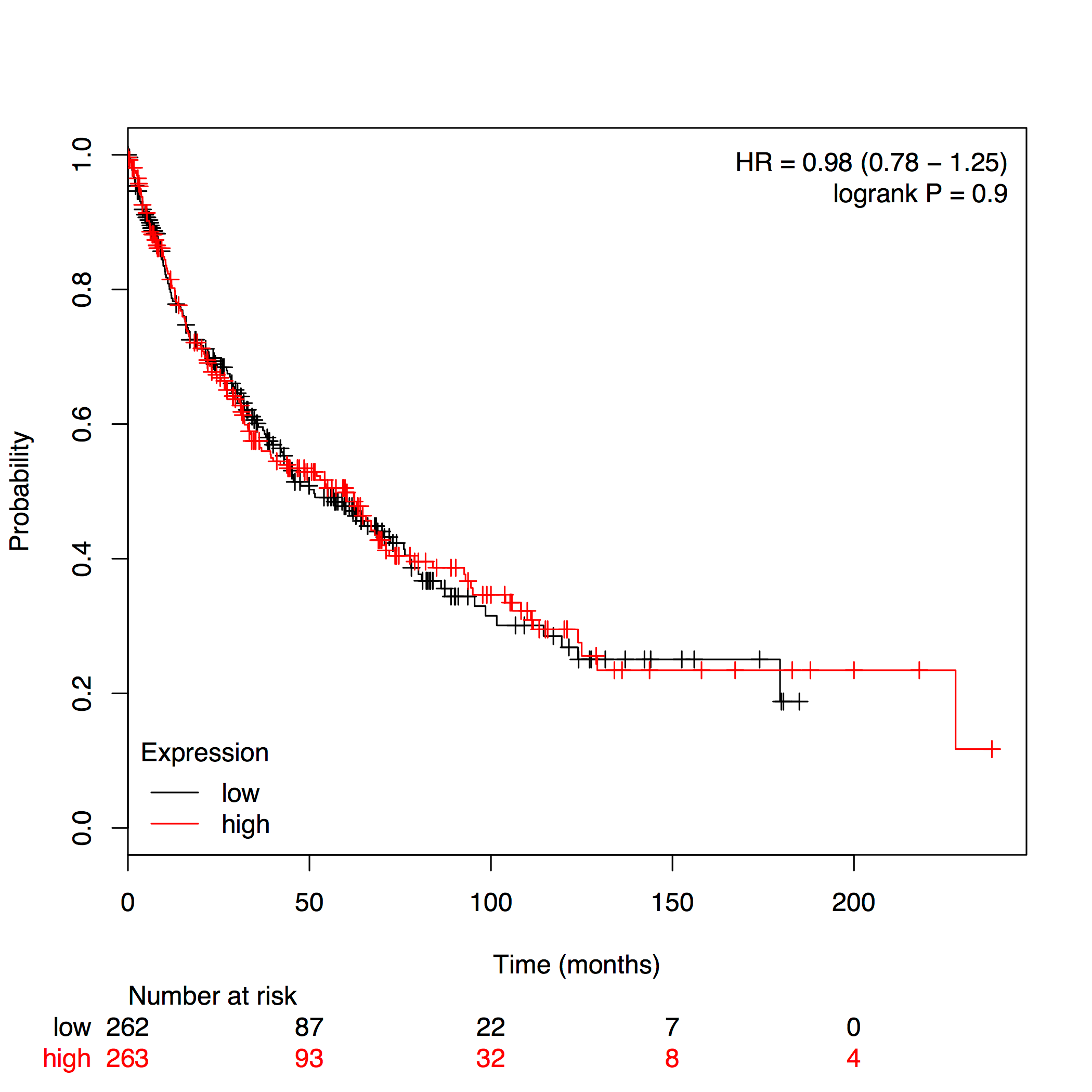

Supplement: S2 Fig — Survival analysis of SCC patients expressing low and high levels of MCT1 (median cutoff). (TIF) [file pone.0142162.s002.tif]

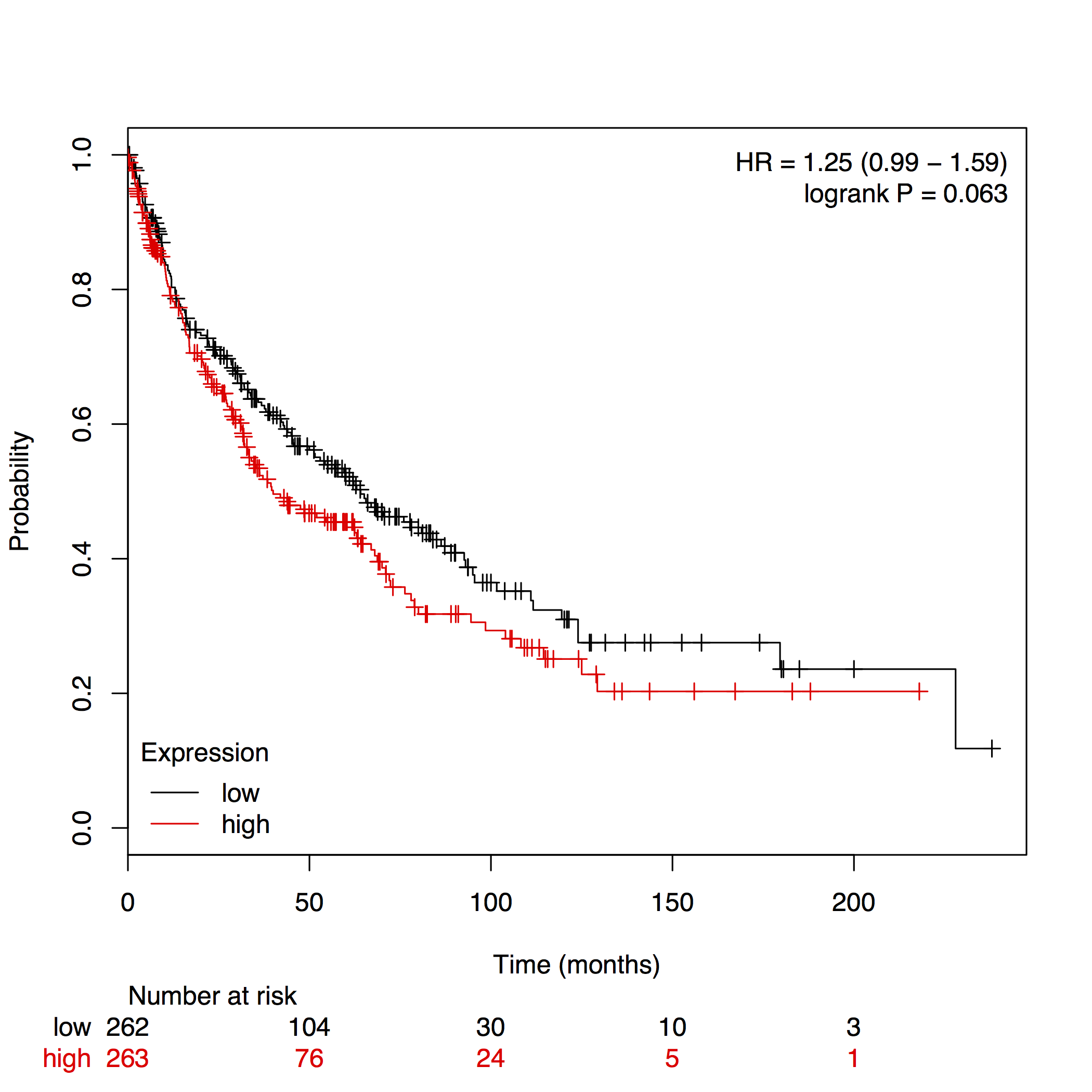

Supplement: S3 Fig — Survival analysis of SCC patients expressing low and high levels of GLUT1 (median cutoff). (TIF) [file pone.0142162.s003.tif]
